# Elucidation of the Relationships between H-Bonding Patterns and Excited State Dynamics in Cyclovalone

**DOI:** 10.3390/molecules190913282

**Published:** 2014-08-28

**Authors:** Marco Lamperti, Angelo Maspero, Hanne H. Tønnesen, Maria Bondani, Luca Nardo

**Affiliations:** 1 Department of Science and High Technology, University of Insubria, Via Valleggio, 11, 22100 Como, Italy; E-Mails: marco.lamperti@uninsubria.it (M.L.); angelo.maspero@uninsubria.it (A.M.); 2 School of Pharmacy, University of Oslo, P.O. Box 1068 Blindern, 0316 Oslo, Norway; E-Mail: h.h.tonnesen@farmasi.uio.no; 3 Institute of Photonics and Nanotechnology, Natl. Research Council, Via Valleggio, 11, 22100 Como, Italy; E-Mail: maria.bondani@uninsubria.it; 4 Department of Health Sciences, University of Milano Bicocca, Via Cadore, 48, 20900 Monza, Italy

**Keywords:** cyclovalone, curcuminoid, photosensitizer, infrared and UV-Vis absorption, fluorescence, H-bonding, photodegradation

## Abstract

Cyclovalone is a synthetic curcumin derivative in which the keto-enolic system is replaced by a cyclohexanone ring. This modification of the chemical structure might in principle result in an excited state that is more stable than that of curcumin, which in turn should produce an enhanced phototoxicity. Indeed, although curcumin exhibits photosensitized antibacterial activity, this compound is characterized by very fast excited-state dynamics which limit its efficacy as a photosensitizer. In previous works we showed that the main non-radiative decay pathway of keto-enolic curcuminoids is through excited-state transfer of the enolic proton to the keto-oxygen. Another effective deactivation pathway involves an intermolecular charge transfer mechanism occurring at the phenyl rings, made possible by intramolecular H-bonding between the methoxy and the hydroxyl substituent. In this paper we present UV-Vis and IR absorption spectra data with the aim of elucidating the intramolecular charge distribution of this compound and its solvation patterns in different environments, with particular focus on solute-solvent H-bonding features. Moreover, we discuss steady state and time-resolved fluorescence data that aim at characterizing the excited-state dynamics of cyclovalone, and we compare its decay photophysics to that of curcumin. Finally, because during the characterization procedures we found evidence of very fast photodegradation of cyclovalone, its photostability in four organic solvents was studied by HPLC and the corresponding relative degradation rates were calculated.

## 1. Introduction

**Figure 1:**
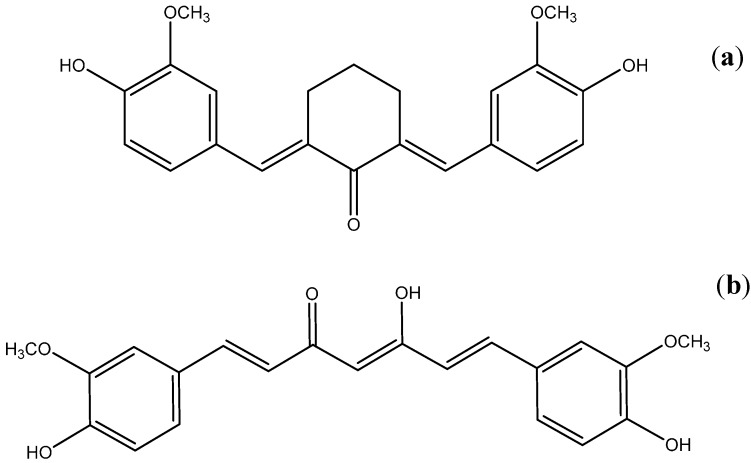
Structure of (**a**) Cyclovalone (CYV) and (**b**) Curcumin (CURC).

This work is part of our series of papers aimed at identifying curcumin (CURC) analogues exhibiting enhanced phototoxicity with respect to the parent compound. In this article we present a detailed spectroscopic characterization of cyclovalone (CYV), a synthetic CURC derivative in which the keto-enolic system is substituted by a saturated cyclohexanone ring (Figure 1a), and provide preliminary considerations on the suitability of CYV as a candidate curcuminoid with optimized photosensitizing properties. 

CURC, Figure 1b, the dietary pigment extracted from turmeric, is considered a very promising natural drug substance, although the poor water solubility of the compound at acidic pH values and its fast hydrolysis at neutral and basic ones represents an issue to be overcome prior to formulation of any CURC-based drug [1]. A constantly increasing number of publications have shown that CURC displays notable effects, not only as an anti-inflammatory compound [2,3,4] and a potent antioxidant [5,6,7,8], but also as a chemopreventive [9,10] and chemotherapeutic [9,11,12] agent. Moreover, it seems to have potential in the treatment of Alzheimer’s disease [13] and cystic fibrosis [14], as well as being considered a model substance for the treatment of HIV-infections and an immune-stimulating agent [15]. Most of the biochemical reactions responsible for its therapeutic properties are believed to involve the highly reducing keto group and the phenyl rings, where the *para*-methoxy and the *meta*-hydroxyl substituents come together to constitute a very peculiar local charge distribution. Some authors have suggested that intramolecular H-bonding between these moieties is essential for several of the observed biochemical properties [16,17]. Formation of this H-bond has been demonstrated in CURC crystals [18,19], but not yet in solution. In any case it is ascertained that the presence of both the hydroxyl and the methoxy groups are indispensable for the pharmaceutically relevant reactions conferring upon CURC its antioxidant [8,20,21,22], metal chelating [23] and radical scavenging [23] properties, as well as to permit the interaction with the nuclear cell factor κB leading to the *in vitro* inactivation of the latter biomolecule, which might be involved in the chemopreventive efficacy of CURC [17]. Indeed, demethoxy and bis-demethoxy curcumin, the other diarylheptanoid constituents of turmeric, are much less effective than CURC and almost inactive, respectively, with respect to the latter important biological properties. Another interesting property of CURC involves its phototoxicity, which has been recently demonstrated on both bacterial and mammalian cells [24,25,26,27,28,29,30,31,32,33,34,35]. Because of the abovementioned instability and/or slight solubility of both CURC and most CURC analogues in aqueous environments [1], performing standard photochemical activity tests, e.g., measurements of the triplet state quantum yield, determination and quantitation of the reactive oxygen species (ROS) produced, assessment of the delivered photo-damage in cells in terms of both elucidation of which biomolecules are oxidized and estimation of the overall photo-induced cell death, is not straightforwardly feasible on bare curcuminoids in an aqueous environment, but rather requires incorporation of the tentative active principle in water soluble drug carriers, e.g., surfactant micelles or cyclodextrins [1]. Selecting an effective carrier is not always a trivial task, thus it should be attempted only for the most promising drug candidates. Furthermore, the photophysics of the drug incorporated in a carrier is not comparable to that of the drug directly dissolved in water, mainly because the microenvironment experienced by the drug within the carrier is typically hydrophobic. Moreover, because carriers with cavities endowed with different polarity and H-bonding properties are available, assessment of the differential behavior of the curcuminoid under trial with respect to environmental changes is a valuable tool in speeding up the selection of optimal carriers.

In a series of previous works by our group, the excited-state dynamics of CURC were investigated [36] and compared to those of a number of phenyl-substituted curcuminoids [37,38,39]. Our studies resulted in a self-consistent model of CURC decay mechanisms from *S*_1_, in which the relative probability and the kinetics of the different deactivation pathways of this tentative photosensitizer appear to be dictated by its intra- and inter-molecular H-bonding patterns. We showed that the fastest non-radiative *S*_1_-decay process for native CURC is an excited-state intramolecular proton transfer (ESIPT) between the hydroxyl- and the keto-group of CURC closed *cis*-enol conformer. Furthermore, we demonstrated that ESIPT can take place only if an intramolecular H-bond (keto-enolic intramolecular H-bond, KEIHB) is formed between the ketone and enol carbonyl groups, and that the ESIPT rate is the faster the more unperturbed is the H-bond, e.g., in a non-polar environment. Both the polarity of the solvent and its capacity to form inter-molecular H-bonds influence the KEIHB stability. Negative residual charges on the carbonyl oxygen inhibit the KEIHB formation and the π-system delocalization, whereas inter-molecular hydrogen bonding perturbs the KEIHB. Hydrogen bond donating solvents interact with the keto moiety, while hydrogen bond acceptors interact with the enol proton. This results in slower, solvent-rearrangement moderated ESIPT. The latter occurs in case a solvated *trans* enol or open *cis* enol molecule isomerizes, upon desolvation, to the closed *cis* enol conformer while in the *S*_1_ state, and then decays to *S*_0_ by means of ESIPT. We also demonstrated [39] that the ESIPT rate in an inert environment, and thereby the strength of the unperturbed KEIHB, correlates with the electron withdrawing/donating properties of the phenyl substituents. Another very efficient decay mechanism takes place in H-bonding solvents, and involves charge transfer interactions between the phenyl substituents and solvent molecules. The latter mechanism is unique for CURC and does not take place in any of the other differently phenyl-substituted curcuminoids we tested [38,39]. Finally, a reketonization dynamics was observed for all the tested phenyl-substituted curcuminoids, involving excited-state cis/trans isomerization and subsequent decay through transfer of the non-H-bonded enol proton to the carbonyl carbon.

Because any *S*_1_ decay mechanism competing with the pathway(s) inducing phototoxicity reduces the photosensitizing potency, it would be desirable to obtain a CURC analogue conserving the main (photo)biological properties of the parent compound, but endowed with enhanced *S*_1_ stability. In previous studies we validated the approach of stabilizing *S*_1_ by varying the phenyl substituents to maximize electron transfer from the aromatic rings to the keto-enol system. We also attempted to perturb the symmetry and/or the semi-aromatic character of the keto-enol system by addition of a substituent at the carbonyl group [40], and indeed a notable reduction of ESIPT efficiency and a shift of the keto-enolic equilibrium towards the diketo conformer were obtained. However, the acidic moiety which was attached to the carbonyl group strongly interacted with solvent molecules in H-bonding environments, and a charge transfer mechanism, which was not observed for either CURC or other carbonyl non-substituted curcuminoids, was introduced by the above modification. Moreover, the relatively fast mechanism of decay through reketonization was observed in all the tested environmental conditions, due to the comparable stability of the keto-enol and diketo conformers.

By virtue of its chemical structure, CYV, the compound to which the present study is devoted, promises enhanced phototoxic activity. Indeed, in such a structure neither the ESIPT nor the reketonization mechanisms dominating the decay dynamics of all the previously evaluated keto-enolic curcuminoids can take place. We were able to find only a few papers dedicated to the chemistry of cyclopentanones and cyclohexanones, in which the spectral and intramolecular charge distribution properties of either CYV or chemically related compounds are investigated [41,42,43,44,45]. However, it is established that CYV conserves many of the most significant ground-state biological properties of CURC, including the anti-inflammatory (*i.e.*, cyclooxygenase inhibitory) and the anti-HIV activity [46,47].

In this work, the H-bonding properties of CYV were investigated by means of infrared (IR) spectroscopy and were correlated to the compound’s photophysical properties and excited-state dynamics, as determined by ultraviolet-visible (UV-Vis) absorption spectroscopy, steady-state and time-resolved fluorescence measurements, ROS generation efficiency and photodegradation studies.

## 2. Results and Discussion

### 2.1. IR Spectroscopy and H-Bonding Patterns

IR spectroscopy has proven to be a versatile tool to investigate inter- and intra-molecular interactions in terms of hydrogen bonding. With the aim to evaluate the H-bonding patterns of CYV, IR spectra of the compound were measured in the solid state and in several organic solvents differing in polarity and H-bond formation affinity. Here we limit our analysis only to a short discussion of the OH stretching region of the infrared spectra of CYV. 

In the solid state the OH-stretching band appears at 3430 cm^−1^, independently of the medium in which the compound is embedded (see Table 1). The shift from the ≈3700 cm^−1^ value characteristic of free OH-stretching suggests that the phenyl OH groups are involved in an H-bond of moderate strength. The independence from the bulk in which CYV is inserted suggests that the latter H-bond is intramolecular in nature. We thus ascribe the OH-stretching band shift to interaction of the *para*-hydroxyl substituents with the nearby *meta*-methoxy substituents of the CYV aromatic rings, in analogy to what was observed for CURC by crystallography [18,19].

**Table 1:** Solid-state and solution O-H stretching of CYV.

	ν(O-H) cm^−1^		
	*Free*	*Intra H-Bond*	*Inter H-Bond*
KBr disk		3370	
Nujol		3375	
Thin film		3378	
Carbon tetrachloride		3553	
Dichloromethane		3530	
Acetonitrile	3636	3542	3392
Acetone	3620 *	3540 *	3350
Dimethylsulfoxide		3450 *	3080

* Shoulder.

**Figure 2:**
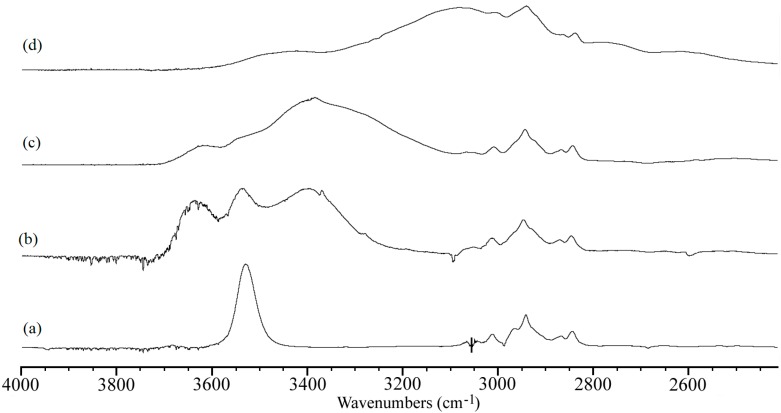
O-H stretching region of the IR-absorption spectra of CYV in solutions of (**a**) dichloromethane; (**b**) ACN; (**c**) acetone; and (**d**) dimethylsulfoxide. The spectrum of carbon tetrachloride was omitted due to the noisiness of the pertaining OH stretching signal.

Due to the poor solubility of CYV in carbon tetrachloride and dichloromethane, the IR spectra resulted quite noisy. However we managed to get neat signals of the OH-stretching band (Figure 2). The spectra recorded in dichloromethane, Figure 2a, and carbon tetrachloride (not shown), two slightly polar and minimally H-bonding solvents, show a single sharp band centred at 3530 cm^−1^ and 3553 cm^−1^, respectively, which denote the O-H stretching vibration to be slightly perturbed by a weak H-bonding interaction.

Because of the very limited reactivity of the solvents under discussion, we propend for attributing the latter interaction to the survival also in solution of the same intramolecular H-bond with the methoxy substituents observed in the solid state. In acetonitrile (ACN), a complex spectral pattern is observed, see Figure 2b. In this very polar, although slightly H-bonding solvent, besides a band centered at 3542 cm^−1^, in a spectral region very similar to that in which the single bands observed in relatively inert solvents (*i.e.*, tetrachloromethane and dichloromethane) fell, we observe two other O-H stretching bands. One of them is around 3639 cm^−1^, the other at 3392 cm^−1^. We attribute the former to the stretching vibration frequency proper of free hydroxyl groups, and the latter to hydroxyl groups interacting with solvent molecules through weak H-bonding interactions. The CN group of ACN is indeed endowed with a slight H-bond accepting character (Kamlet basicity parameter β = 0.31 [48]). In this picture, the free O-H stretching frequency is observed because of non-specific polarity interactions between the hydroxyl (and methoxy) groups of CYV and solvent molecules, being able to disrupt the weak intramolecular H-bond but too unstable to induce a significant shift in the O-H stretching band. Also in acetone, Figure 2c, we observe a similar spectral pattern, but in this solvent the band attributed to the stretching of hydroxyl groups interacting with the solvent by intermolecular H-bond formation (peak at ≈3450 cm^−1^) is much more intense than the others. Indeed, the band at ≈3540 cm^−1^ produced by stretching vibrations of hydroxyl groups involved in intramolecular H-bonds with the methoxy moieties is only perceived as a shoulder of this main, broad band. Both the broadening and the superior relative intensity of the ≈3450 cm^−1^ band with respect to the ≈3540 cm^−1^ band compared to the ACN spectrum suggest that the acetone ketone group is much more reactive as an H-bond acceptor than the CN moiety of ACN, in agreement with the higher Kamlet basicity parameter tabulated for this solvent (β = 0.48 [48]). Finally, in dimethylsulfoxide (DMSO), Figure 2d, the main O-H stretching band is notably downshifted to 3080 cm^−1^, indicating formation of strong intermolecular H-bonds between the hydroxyl groups of CYV and this solvent endowed with high H-bond accepting character (β = 0.76 [48]). Moreover, while the ≈3540 cm^−1^ band is still detectable as a shoulder of the main band, indicating that even in DMSO the hydroxyl-methoxy intramolecular H-bonds are not totally disrupted, the >3600 cm^−1^ band vanishes, suggesting that all the hydroxyl groups are involved in H-bonding interactions, being the latter either intra- or inter-molecular.

### 2.2. UV-Vis Absorption Spectroscopy

The UV-Vis absorption spectra of CYV are dominated by two UV bands, one peaking at ≈250 nm and the other with maximum around 370 nm. Roughly the same values were observed by Issa and coworkers [43] for a series of diarylcyclohexanones, including CYV, in other organic solvents. In the latter article the authors attributed the short-wavelength band to the π-π* transition of the aromatic systems, while the long wavelength band was ascribed to an intramolecular electron transfer from the highest filled energy level of the aromatic system to the lowest vacant level of the keto group. Indeed, the charge transfer character of this electronic transition is supported by the higher intensity and broadening of the corresponding band compared to the short-wavelength band. The position of the absorption peak at ≈250 nm is nearly independent on the properties of the solvent, *i.e.*, 248 nm < λ_Max_ < 254 nm with apparently no correlation to the solvent polarity or H-bonding properties. This indicates the localized nature of the corresponding transition. Conversely, the charge-transfer transition peak exhibits notable dependence from the solvent properties (*vide infra*). It is worth noting that, among the differently phenyl-substituted compounds considered in [43], CYV displays by far the most red-shifted charge transfer band, *i.e.*, the most extensive conjugation between the phenyl rings and the central cyclohexanone. Since from the detailed explanation of the charge transfer mechanism offered in [43] it results apparent that the more the phenoxyl proton is depleted of its electronic charge, the more efficient is electron transfer from the phenyl to the diketone, and consequently the more extensive is the charge transfer band red shift, this observation supports the formation of H-bonding interactions between the hydroxyl and methoxy groups, which is also suggested by the IR data.

**Figure 3:**
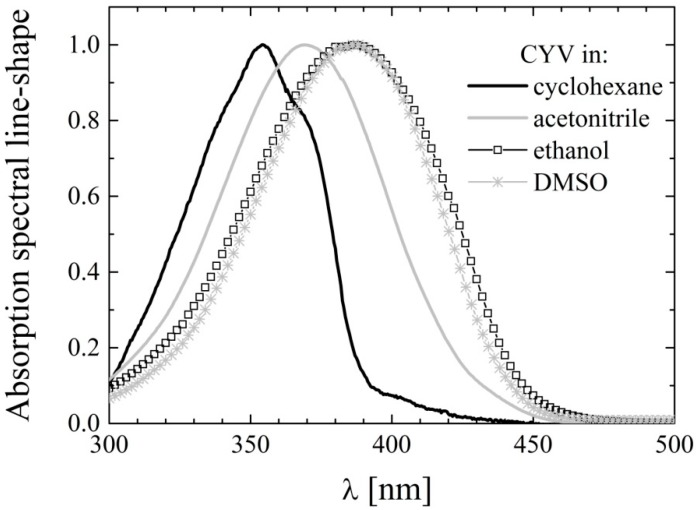
Charge-transfer absorption bands of CYV in selected solvents: cyclohexane (black line), acetonitrile (gray line), ethanol (squares) and dimethylsulfoxide (stars).

The absorption maxima of the charge transfer band, λ_Abs_, of CYV in the different solvents are listed in Table 2. For the purpose of straightforward comparison, the corresponding values for the maximally red-shifted absorption band of CURC (data replicated from [36]) are also reported. Some representative absorption spectra of CYV are displayed in Figure 3. In all solvents, including cyclohexane in which CURC exhibited two well defined peaks, the ≈370 nm absorption band of CYV was broad and essentially structure-less.

**Table 2:** Absorption maxima of the charge transfer band of CYV (λ_Abs_) and main absorption band of CURC (λ_CURC_) in selected solvents.

Solvent		λ_Abs_ (nm)	λ_CURC_ (nm)
**Non Polar**	Cyclohexane	355	408,429
**Polar weakly H-bonding**	Chloroform	372	419
	Ethyl acetate	367	419
	Acetone	370	420
	Acetonitrile	370	419
**Strong H-bond acceptors**	DMFA	383	431
	DMSO	386	434
**Alcohols**	Ethanol	387	430
	Methanol	385	423

In previous works of ours [36,37,38,39] the two peaks resolved for CURC and other keto-enolic curcuminoids in cyclohexane were ascribed to the coexistence of enol and diketo conformers in that solvent. The lack of structure for CYV, which does not exhibit keto-enol tautomerism, supports our previous interpretations. The solvent dependency of λ_Abs_ is very similar for CYV and CURC. Both compounds undergo notable (≈10 nm) red shift as the environment is changed from non-polar to polar and further bathochromic shift (≈20 nm compared to cyclohexane) when dissolved in H-bonding solvents. However, the λ_Abs_ values are as much as 50 nm blue shifted for CYV than for CURC. This reflects the decrease in intramolecular charge conjugation caused by substitution of the conjugated double-bonds of the keto-enol moiety in CURC with the saturated bonds of the cyclohexanone ring in CYV. Indeed, charge conjugation, which is likely to be extended over the whole molecule in the case of keto-enolic CURC analogues, is limited to the charge flux from the phenyl rings to the double-bonds of the aliphatic chain in CYV. As a consequence, the λ_Abs_ values shifts towards those reported for half-curcumin, exhibiting its main absorption band around 320–340 nm [49,50]. As we previously noted for CURC [38,39], and was postulated by Issa *et al.* [43] on the basis of the sole datum of absorption in ethanol, the λ_Abs_ shift cannot be accounted for by a mere dependence on non-specific polarity-driven solvation equilibrium. Conversely, the additional red-shift observed in all the solvents having H-bonding ability, the minor variations in λ_Abs_ recorded for solvents of very different polarity but similarly weak H-bonding abilities such as e.g., ACN and ethyl acetate, and the major shift observed by changing from ACN to methanol and dimethylformamide (DMFA), solvents of very similar polarity and very different H-bonding character, compel to conclude that solute-solvent H-bonding interactions are of major importance for the spectral properties.

From the pharmaceutical standpoint, it should be noted that the penetration depth of UVA light in tissues is not very different to that of green light, the former being capable of reaching the deepest dermis layers and the latter barely skimming the most superficial layers of subcutaneous adipocytes. For this reason, the observed 50 nm blue-shift in the absorption maximum of CYV with respect to that of CURC is not much penalizing in terms of their photopharmaceutical properties. Indeed, photo-therapeutic applications of all the curcuminoids we have tested so far, displaying absorption peaks below 440 nm, are essentially confined to the treatment of pathologies of the epidermis and of the oral cavity, or to endoscopic illumination.

### 2.3. Steady-State Fluorescence

The fluorescence emission spectra of CYV in the same solvents used for UV-Vis analysis were recorded by taking advantage of the enhanced sensitivity of the PTI photomultiplier tube. The fluorescence emission was generally very weak, with the exception of alcohols in which CYV emits moderate fluorescence. The fluorescence emission maxima are tabulated in Table 3.

**Table 3:** Fluorescence emission maxima of CYV and CURC in selected solvents. Excitation @ λ_Abs_.

Solvent		λ_Abs_ (nm) (Φ_Fluor_)	λ_CURC_ (nm) (Φ_CURC_)
**Non polar**	Cyclohexane	481 ([4 ± 1] × 10^−5^)	502, 471, 443 (0.006 ± 0.003)
**Polar weakly H-bonding**	Chloroform	478	503
	Ethyl acetate	487	494
	Acetone	502	510
	Acetonitrile	496 ([4 ± 1] × 10^−5^)	521 (0.156 ± 0.003)
**Strong H-bond acceptors**	DMFA	514	536
	DMSO	511 ([4.4 ± 0.5] × 10^−4^)	550 (0.026 ± 0.002)
**Alcohols**	Ethanol	509 ([1.2 ± 0.1] × 10^−3^)	553 (0.033 ± 0.003)
	Methanol	518 ([1.4 ± 0.2] × 10^−3^)	566 (0.028 ± 0.002)

**Figure 4:**
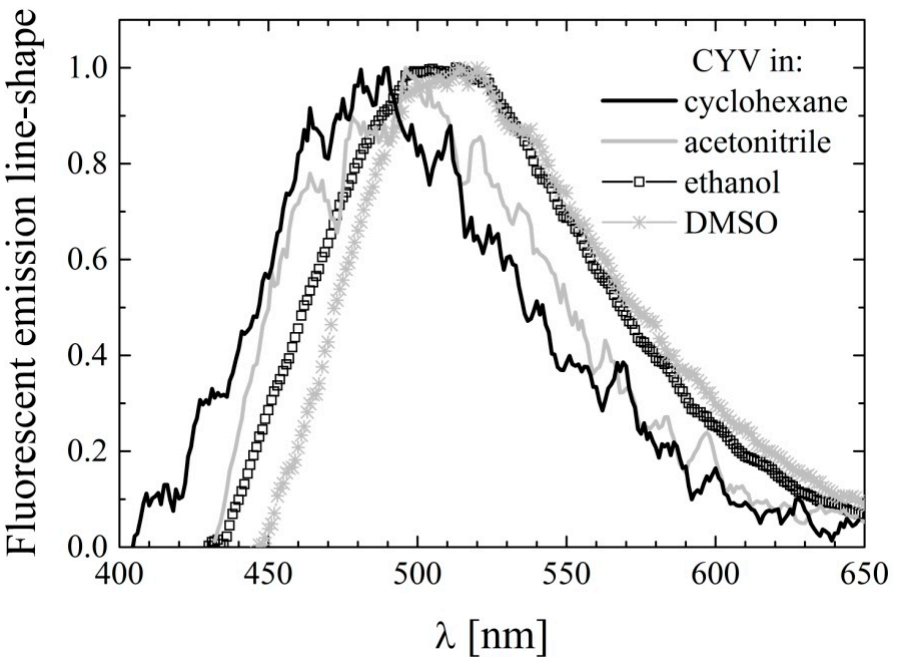
Fluorescence emission spectral line-shapes of CYV in selected solvents: cyclohexane (black line), acetonitrile (gray line), ethanol (squares) and dimethylsulfoxide (stars).

The emission maximum is generally blue-shifted and less solvent dependent in CYV compared to CURC. This suggests a more localized and less mobile electronic charge in CYV, which is consistent with its molecular structure in which the conjugation between the two phenyl rings through resonance of the heptadiene double-bonds, which occurred in CURC, is prevented by insertion of the saturated cyclohexanone ring. The fluorescence spectral line-shapes of CYV in selected solvents are shown in Figure 4. In all of the tested solvents they appear structure-less, even in cyclohexane, where CURC exhibited three emission peaks. This further supports our interpretation of the bands observed for CURC to be a result of keto-enol tautomerism [36].

In the non-alcoholic solvents we only managed to acquire very noisy spectra, as inferable from Figure 4, so that it was difficult to determine the fluorescence quantum yield values for all these solvents. For this reason one solvent representing each category was chosen, *i.e.*, cyclohexane (non-polar), ACN (polar non-H-bonding), and DMSO (H-bond acceptor). The fluorescence quantum yield, Φ_Fluor_, was calculated upon averaging over ten parallel samples. In spite of this large averaging, the Φ_Fluor_ values, which are also reported in Table 3, are affected by quite large errors. In addition, we determined the Φ_Fluor_ values in alcohols (*i.e.*, methanol and ethanol) upon averaging over three parallels. In any case, one can observe that Φ_Fluor_ is minimal in non-polar and polar weakly-H-bonding environment and only slightly increases in polar H-bond accepting solvents, while it steeply increases in solvents endowed with H-bond-donating character. A very similar behavior has been observed in 2,5-diarylidenecyclopentanones [44], a class of compounds very similar to 2,5-diarylidenecyclohexanones like CYV. With the support of computational calculations, this particular dependence of fluorescence on solvent properties has been explained in terms of the relative energy of the *S*_1_ and *S*_2_ states [44]. According to the Authors, the *S*_1_ state is nπ* and *S*_2_ is ππ* in the gas phase (and thus most likely in inert environment). As explained in details in [51], radiative decay from nπ* is effectively quenched by intersystem crossing to the ππ* triplet lying between *S*_0_ and *S*_1_, because of the strong spin-orbit coupling existing between singlet and triplet states of different orbital configuration. However, nπ* and ππ* orbitals are differently perturbed by solvation. While the nπ* states shift to higher energies at increasing solvent reactivity (*i.e.*, polarity and H-bonding ability), ππ* states are stabilized by interaction with the solvent. It should also be noted that in the first ππ* excited singlet state of 2,5-diarylidene-cyclopentanones, and thus likely also of 2,5-diarylidene-cyclohexanones, electronic charge is strongly withdrawn from the phenyl rings to the ketone moiety. It is apparent that H-bond-donating solvents have optimal interaction with the ketone residual negative charge, thus stabilizing the ππ* compared to the nπ* configuration more effectively than the other solvents. As a result the first two excited singlet states are probably inverted in these solvents (*i.e.*, *S*_1_ becomes ππ* and *S*_2_ becomes nπ*). Because the spin-orbit coupling is much looser between singlet and triplet orbitals of equal configuration, intersystem crossing is drastically reduced, and Φ_Fluor_ increases, in alcohols. It should be noted that the nπ*–ππ* energy gap in the gas phase is the smaller the higher the conjugation between the phenyl ring and the ketone group. In the 2,5-diarylidenecyclopentanones studied in [44], the energy gap is calculated to be 8,400 cm^−1^ for 2,5-(dibenzylidene)cyclopentanone, and it is roughly the half for 2,5-bis-(3-phenylallylidene)cyclopentanone. In the same work it is experimentally shown that 2,5-(dibenzylidene)cyclopentanone is non-fluorescent also in alcohols (*i.e.*, solvent H-bonding to the ketone group is not sufficient to reverse the nπ*–ππ* order). Only for 2,5-bis-(3-phenylallylidene)cyclopentanone a solvent dependence of Φ_Fluor_ similar to the one we observe for CYV is reported. It thus seems that the presence of both *meta*-hydroxyl and *para*-methoxy substituents in the aromatic rings has an effect on phenyl-ketone conjugation (*i.e.*, on the electron donating character of the phenyl) comparable to that obtained by addition of a conjugated double bond in the aliphatic chain.

### 2.4. Photodegradation Studies

**Figure 5:**
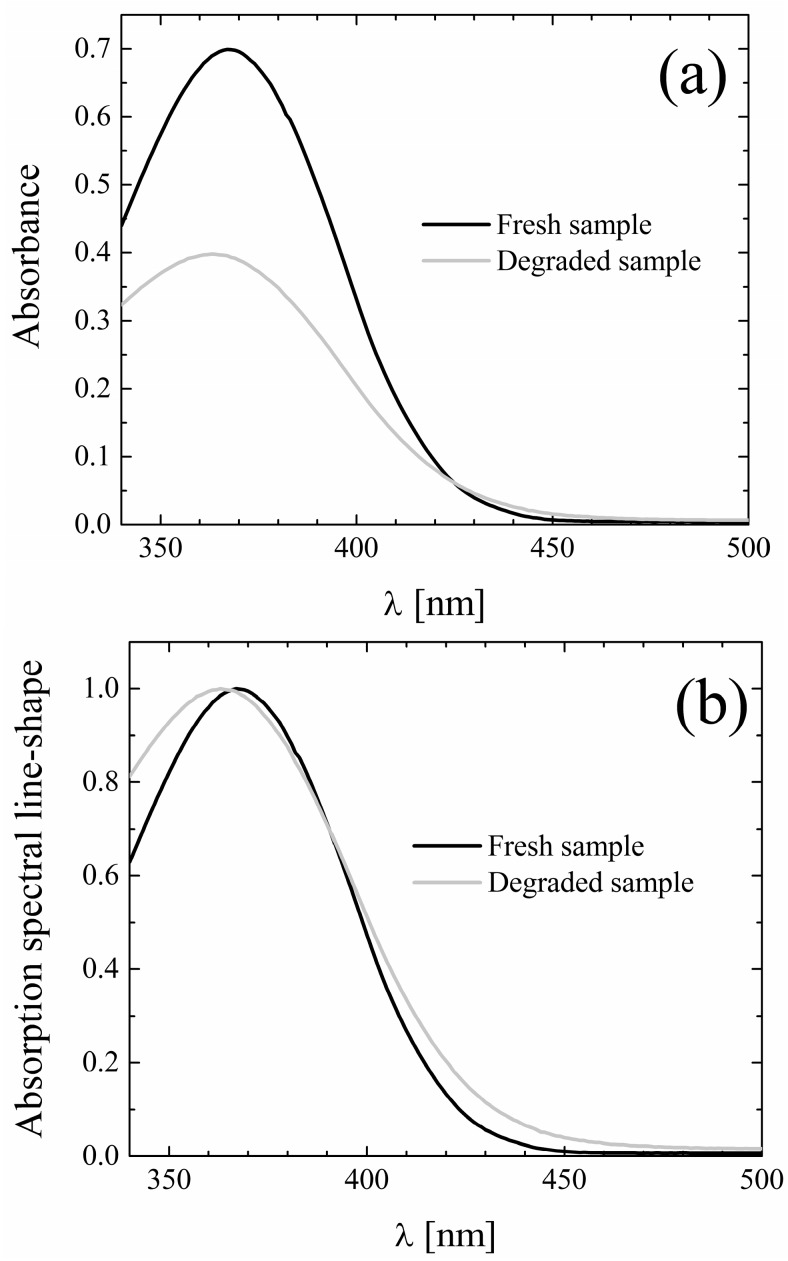
(**a**) Absolute and (**b**) Peak-normalized absorption spectra of fresh (black) and photodegraded (gray) CYV in acetonitrile. In the figure we focus on the modifications experienced by the charge-transfer band, those exhibited by the phenyl π-π transition band at 250 nm are similar.

**Figure 6:**
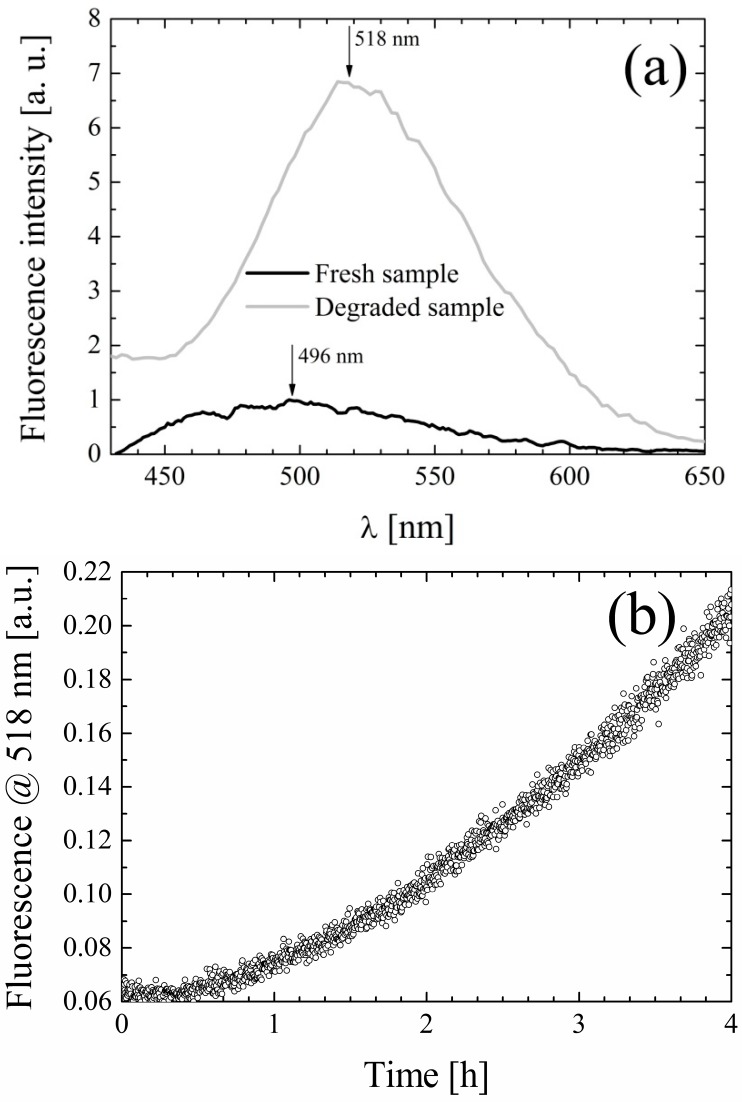
(**a**) Fluorescence emission spectra of fresh (black) and photodegraded (gray) CYV in acetonitrile; (**b**) Increment of fluorescence at 518 ± 2 nm in time upon irradiation at 370 ± 2 nm with ≈400 μW/cm^2^ intensity.

During the experiments it was observed that exposure of the samples to daylight induced the appearance of a yellow-green emission visible to the eye, which was not present in freshly prepared samples. Based on the knowledge that curcuminoids are to a various extent subject to photodegradation [36,37,38,39], a photostability study of CYV was conducted. Samples were prepared in selected solvents (*i.e.*, chloroform, ACN, ethanol and methanol) and divided into three aliquots. The first was stored in the dark overnight, the second was used to acquire absorption and fluorescence emission spectra within <30 min from preparation, and the remaining solution was exposed to the lab light overnight. The spectra obtained from freshly prepared samples and from samples kept overnight in the dark were comparable within the experimental errors. The samples submitted to prolonged light exposure showed, in all the above-mentioned solvents: (i) hypochromic and broadened absorption bands (ii) a notable increase and a slight red shift of the fluorescence emission. Hereby we focus only on the data obtained in ACN, for the sake of brevity and clarity, but the qualitative behavior described for CYV in ACN seems to be solvent-independent. In Figure 5 we report absorption data for the fresh and photodegraded sample dissolved in ACN. Moreover, to make the band-broadening more visible, we zoom on the portion of the absorption spectrum characterized by the charge-transfer absorption band. The corresponding spectra obtained for CYV dissolved in ethanol, including also the π-π transition band, are enclosed for the sake of comparison as Supplementary Materials, Figure S1 . The emission spectra for the same solutions are shown in Figure 6a. The emission peak shifts from 496 nm to 518 nm and the fluorescence intensity increases about seven-fold.

To conclude this preliminary characterization of the effects of light on the spectral properties of CYV we prepared a new sample in ACN and recorded the variation of fluorescence at 518 ± 2 nm in time upon excitation at 370 nm (excitation bandwidth 4 nm, lamp band-integrated spectral intensity ≈400 μW/cm^2^). The results, plotted in Figure 6b, show that substantial alteration of the fluorescence signal (*i.e.*, measurable photodegradation) occurs within hours even at this low illumination rate, comparable to that of daylight in the same band.

In the Suntest CPS, under the experimental conditions fully described in the Experimental Section, the following degradation half-lives were obtained for CYV dissolved in the solvents of above: Chloroform: 12.8 min; ACN: 12.6 min; Methanol: 17.8 min; Ethanol: 17.8 min. In methanol and ACN also the photodegradation quantum yield, Φ_Degr_, was measured. The values were roughly one order of magnitude larger than those of CURC (*i.e.*, Φ_Degr,CYV_ = 0.22 ± 0.11 and Φ_Degr,CURC_ = 0.061 ± 0.011 in ACN, while Φ_Degr,CYV_ = 0.176 ± 0.034 and Φ_Degr,CURC_ = 0.021 ± 0.010 in methanol). Five photodegradation products were isolated in ethanol by TLC and their UV-Vis absorption spectra were recorded. They are enclosed as Supplementary Material, Figure S2. The degradation products exhibit their most red-shifted peak wavelength within the CYV charge-transfer absorption band. This result agrees with the empirically observed slight broadening of the CYV absorption band, without onset of other well distinguishable absorption peaks, upon light exposure.

### 2.5. Time-Resolved Fluorescence

The fluorescence decay distributions of CYV in all the solvents mentioned above were reconstructed by means of Time-Correlated Single-Photon Counting (TCSPC) upon excitation at 420 nm by the second harmonic output of a Ti:sapphire laser (see Experimental Section) [52]. By taking advantage of the single photon sensitivity of our detector we managed to acquire very neat decay histograms in spite of the very low Φ_Fluor_ of the compound, with the exception of the sample dissolved in cyclohexane, for which the absorption band is too blue-shifted for the electronic transition to be induced by visible light. The decay parameters are reported in Table 4.

**Table 4:** Decay parameters of CYV in selected solvents.

Solvent	τ_1_ [ps] (_A1_)	τ_2_ [ps] (A_2_)	τ_3_ [ps] (A_3_)	τ_4_ [ps] (A_4_)	τ_5_ [ps] (A_5_)	τ_Av_ [ps]
Cyclohexane (exc. 355 nm)	-	188 ± 5 (0.89)	-	-	2416 ± 68 (0.11)	433
Chloroform	11 ± 2 (0.84)		203 ± 4 (0.14)	807 ± 42 (0.02)	2194 ± 212 (<0.01)	391 (75)
Ethyl acetate	-	-	-	1074 ± 9 (0.57)	4088 ± 38 (0.43)	2370
Ethyl acetate (exc. 355 nm)	-	-	-	1004 ± 40 (0.56)	3804 ± 370 (0.44)	2236
Acetone	34 ± 2 (0.80)	-	-	965 ± 6 (0.09)	3852 ± 23 (0.11)	2553 (538)
Acetonitrile	10 ± 1 (0.86)		222 ± 0 (0.14)	-	2795 ± 160 (<0.01)	394 (67)
DMFA	24 ± 1 (0.58)	-	190 ± 12 (0.08)	765 ± 8 (0.19)	4500 ± 12 (0.15)	1989 (849)
DMSO	38 ± 1 (0.71)	-	208 ± 2 (0.26)	1021 ± 57 (0.02)	5032 ± 91 (0.01)	430 (152)
Ethanol	35 ± 2 (0.63)	-	232 ± 3 (0.14)	1340 ± 15 (0.03)	5419 ± 19 (0.20)	3126 (1179)
Ethanol (exc. 355 nm)	36 ± 1 (0.78)	-	248 ± 6 (0.12)	1394 ± 30 (0.06)	5500 ± 100 (0.04)	361 (1515)
Methanol	23 ± 1 (0.82)	-	223 ± 2 (0.16)	1374 ± 134 (0.01)	5470 ± 173 (0.01)	578 (123)
Methanol (exc. 355 nm)	18 ± 2 (0.74)	-	227 ± 18 (0.10)	1350 ± 46 (0.08)	5500 ± 150 (0.08)	2195 (584)

In the same Table we report the decay parameters obtained for CYV in cyclohexane upon excitation at 355 nm, *i.e.*, at the absorption peak wavelength, with the third harmonic of a Nd:VAN laser (see Experimental Section) [53]. Unfortunately, the efficiency of out-of-cavity generation of the third harmonic is very low, and we only managed to get <100 μW average excitation power. Hence, repetition of TCSPC measurements in all the solvents was prevented due to the very long (several hours) acquisition times, which in turn might undermine both the system electronic stability and the compound integrity against photodegradation by lab light. However, in order to investigate the dependence of the decays on excitation wavelength, we also acquired decay patterns upon excitation at 355 nm of CYV dissolved in the alcoholic solvents, where the fluorescence quantum yield was high enough to warrant reconstruction of sufficiently smooth decay histograms in reasonable times (<1 h), and in ethyl acetate, where photodegradation appeared less severe than in the other solvents (*vide infra*). The decay parameters derived from the fit of these decays are also reported in Table 4, under the corresponding values derived from experimental data with excitation at 420 nm, for the sake of straightforward comparison. The decay times obtained by excitation at the two wavelengths are equal in all these three solvents within the experimental errors. This observation allows us to compare the cyclohexane data with those obtained in the other solvents upon excitation at a longer wavelength.

**Figure 7:**
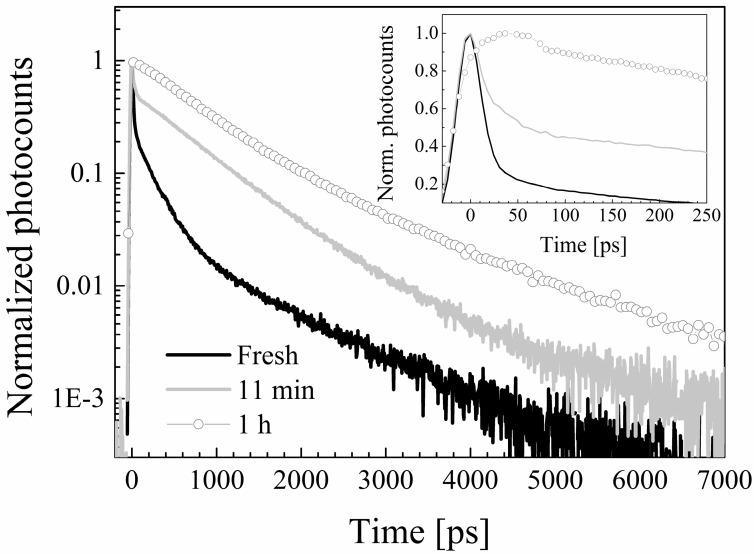
Fluorescence decay patterns of a freshly prepared CYV solution in chloroform (black line), and of the same solution after 10 min (gray line) and one hour (dots) of exposure to the laser beam.

In all the solvents except cyclohexane and ethyl acetate the decay patterns are dominated by a very fast transient with time constant <40 ps (τ_1_ in the Table). Moreover, by re-acquiring TCSPC data after illumination with the Ti:sapphire laser for 10 min and after additional exposure to the laser light for 50 min, *i.e.*, 1 h of overall exposure (excluded cyclohexane for which we remind that excitation at 420 nm was not suitable to induce electronic excitation), the decays resulted to be time-dependent in all the solvents, with the exception of those pertaining to the compound dissolved in ethyl acetate, where they are essentially unaffected by exposure to prolonged laser excitation. Namely, the above mentioned fast component tended to decrease in amplitude and eventually disappear. In Figure 7 we exemplify the above-described phenomenon by showing the decay distributions obtained in chloroform immediately after sample preparation, after 10 min, and after 1 h of exposure to the laser beam (when the non-degraded sample should amount to only few percent of the initial one). 

We thus ascribe the τ_1_ component to photodegradation dynamics. This attribution is also supported by the fact that the time constant measured in alcohols is longer than the one measured in ACN and chloroform, which indicates slower degradation in alcohols, in agreement with both the corresponding degradation half-life and Φ_Degr_ data. The fact that such a short decay component is not detected in cyclohexane and ethyl acetate, *i.e.*, the least interacting solvents, suggests that solvation has a role in triggering the photodegradation pathways.

The shortest decay component detected in inert environment (*i.e.*, cyclohexane, see τ_2_ in Table 4) has a time constant comparable to that measured for CURC and attributed to re-ketonization. It might thus be ascribed to a non-H-bond facilitated ESIPT process. Exchange of a proton between the aliphatic chain and the keto group was indeed previously hypothesized for similar compounds [45]. Alternatively, excited-state electron transfer might take place from the ketone ring (whereby the phenyl electronic charge is withdrawn upon excitation) back to the aliphatic chain double-bonds [43]. In both instances, solvent interactions with the ketone moiety by either a simple polarity effect or intermolecular H-bonding should slow down the charge transfer mechanism. Indeed, in all the other solvents we observe an excited-state deactivation pathway which occurs on the time scale of approximately 1 ns (τ_4_ in Table 4). We attribute this excited-state deactivation pathway to solvent-rearrangement moderated intramolecular charge transfer. To support this assumption, we observe that the pertaining time constant is the longest in alcohols, *i.e.*, the only solvents which are able of forming intermolecular H-bonds with the ketone group. In H-bonding solvents a faster (<250 ps) decay mechanism is also observed (see τ_3_ in the same Table). The latter is very similar to that observed for CURC in the same solvents and attributed to intermolecular charge/energy transfer from the phenyl rings to H-bonded solvent molecules. Because CYV carries the same phenyl substituents, which are made highly reactive by the specific charge distribution induced by H-bonding interactions between the methoxy and hydroxyl moieties of the aromatic ring (see IR data), we ascribe τ_3_ to the same mechanism. Quite surprisingly, a decay component with lifetime similar to τ_3_ is detected also in the weakly H-bonding solvents chloroform and ACN. Solute-solvent charge transfer interactions were not observed for CURC in the same solvents, but agree with the notable perturbation of the intramolecular H-bond between the hydroxyl and methoxy substituents of the aromatic rings evidenced in ACN by IR spectroscopy (see above). Observation of excited-state solute-solvent charge transfer also in these less reactive solvents for CYV but not for CURC might be due to enhanced mobility of the phenyl residual charge in CYV. Finally, in all the solvents a long-lived component with time constant of ≥2.2 ns in cyclohexane and chloroform and ≥3.9 ns in more polar environments is detected. Such a component is present in sizeable amounts with the exception of chloroform and ACN, where the photodegradation is possibly too severe and we barely resolve traces of this deactivation pathway (relative amplitude <0.01). The pertaining time constant is very similar to that detected, and ascribed to the unperturbed radiative decay of the diketo conformers, in the decays of a curcuminoid in which the keto-enolic equilibrium was notably diketo-shifted [40]. We thus speculate that also for cyclovalone, due to removal of the main deactivation pathway of CURC, *i.e.*, KEIHB-favored ESIPT, a sizeable fraction of the excited molecules decays through unperturbed radiative decay.

The above described excited-state dynamics result in an average excited-state lifetime τ_Av_ which is notably longer for CYV than for CURC in all the tested solvents if the degradation rate is disregarded (see last column in Table 4). However, the irreversible loss of great amounts of CYV by photodegradation reduces the amount of stabilization (see τ_Av_ values in parenthesis in the same column, calculated by including the transient ascribed to photodegradation dynamics), and in any case jeopardizes any application of the non-stabilized compound as a photo-activated drug substance, unless a drug carrier is selected in which CYV microenvironment be similar to that experienced by the drug in cyclohexane and ethyl acetate, *i.e.*, very weakly polar and non-H-bonding.

### 2.6. Spectrofluorimetric Detection of Photosensitized Reactive Oxygen Species Generation

**Figure 8:**
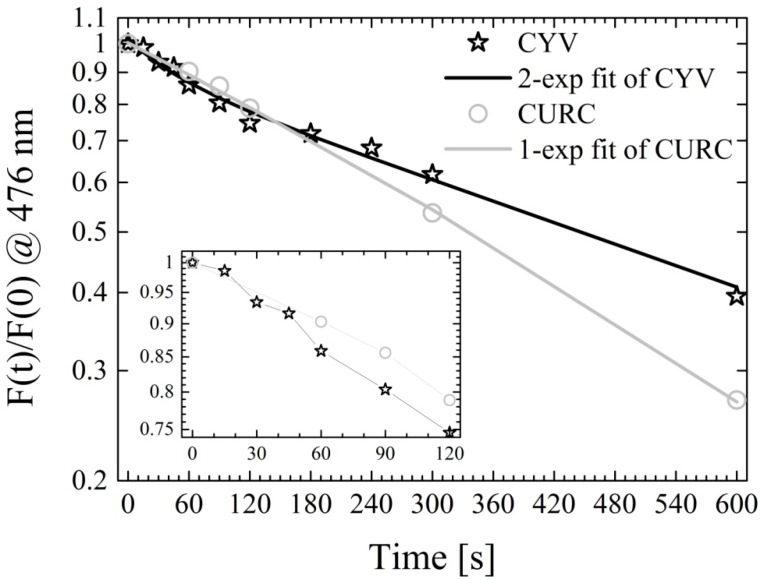
Diphenylisobenzofuran fluorescence as a function of time. Both CYV (black stars) and CURC (grey dots), upon illumination, produce ROS, which induce oxidation of the highly fluorescent compound diphenylisobenzofuran into the non-fluorescent compound *o*-dibenzoylbenzene.

In order to preliminarily assess the potential of CYV as a photosensitizer we exploited the diphenylisobenzofuran fluorescent indicator and measured the photo-induced production of ROS relative to CURC in methanol. Production of ROS, particularly of either singlet oxygen or superoxide radical, induces oxidation of diphenylisobenzofuran into *o*-dibenzoylbenzene, which is non-fluorescent. Thus, a reduction in the fluorescence emission proportional to the concentration of generated ROS is observed [54,55]. The results are shown in Figure 8. 

The ROS production by CYV is initially more efficient than that of CURC (see Figure 8 inset). However, at long light-exposure times this result is reversed. In the effort to interpret this phenomenon on a more quantitative basis, we attempted to fit the fluorescence quenching curves to exponential decays. In the case of CURC the diphenylisobenzofuran fluorescence quenching as a function of illumination time can be optimally fitted to a single-exponential decay with decay rate K_CURC_ = 0.0017 s^−1^ (grey solid line in Figure 8). This entails a single-exponential trend of the cascade production of ROS, too. Conversely, in the case of CYV a two-exponential behavior is observed (black solid line). We interpret this fact as due to the progressive conversion of CYV in photodegradation products which have poor photosensitizing potency. In this picture, at very short light-exposure times we measure the fast diphenylisobenzofuran fluorescence quenching rate K_1_ = 0.02 s^−1^, which is related to the intrinsic rate of ROS production proper of intact CYV, K_ROS_, while at longer light-exposure times the ROS production is dumped by photodegradation, which gradually reduces the concentration of photosensitizer in solution at a rate K_Degr_. Hence, a slower apparent fluorescence quenching rate, K_2_ = 0.0013 s^−1^ is measured, which is related to the difference ΔK = K_ROS_ − K_Degr_. If our interpretation is correct, then the relative ROS generation rate of CYV with respect to CURC, K_ROS,CYV_/K_ROS,CURC_, can be estimated by the ratio K1/K_CURC_ = 11.64.

## 3. Experimental Section

### 3.1. Chemicals and Samples Preparation

CYV (Schweizer Hall, South Plainfield, NJ, USA) was used as received. The solvents used in the present study were divided into the following categories: non-polar (cyclohexane), polar weakly H-bonding (chloroform, ethyl acetate, acetone, ACN), strong H-bond acceptors (DMFA and DMSO), and alcohols (ethanol and methanol). A compendium of selected chemical-physical properties of these solvents can be found in [37]. All the solvents were ≥99.5% pure and were used as received, except for ethyl acetate which was dried over sodium sulfate. Unless otherwise specified, the solutions were prepared immediately before the measurements. 

### 3.2. IR Absorption Spectra

The IR absorption spectra were acquired on a Thermo IS-10 FTIR spectrometer (Thermo Fisher Scientific, Asheville, NC, USA) at a resolution of 1 cm^−1^ in solid state as KBr disks, nujol bulks, and thin films obtained by evaporation, and in carbon tetrachloride, dichloromethane, ACN, acetone and DMSO solutions. The latter solutions were contained in a KBr cell with 0.6 mm path length.

### 3.3. UV-Vis Absorption and Fluorescence Spectra, Fluorescence Quantum Yields

The UV-VIS absorption spectra were measured by a Lambda 2 spectrophotometer (Perkin Elmer, Waltham, MA, USA). Steady-state fluorescence measurements were carried out with the PTI modular Fluorescence System (PTI, London, ON, Canada) described in [36]. The samples, thermostated at 25 ± 0.1 °C, were excited at the absorption peak. The system was equipped with a software (Felix^TM^ for Windows) performing automatic correction of the acquired spectra with respect to the spectral responses of both the excitation lamp and the detector. Fluorescence quantum yields, Φ_Fluor_, were determined from the spectrum integrated fluorescence by using, as a reference value, that of dimethyl-POPOP in cyclohexane: Φ_Ref_ = 0.93 [56]. The Φ_Fluor_ were corrected for differences in peak absorbance and in refractive index of the solvents. The reported values are calculated as the average of ten parallels, with errors given by the pertaining standard deviations.

### 3.4. Fluorescence-Decay Measurements

The fluorescence decays were measured by TCSPC. The used TCSPC setup has ~30 ps time resolution (full width at half maximum of the detected excitation pulse) and is fully described elsewhere [52]. The fluorescence of the solutions, which were contained in a 1 × 1 cm^2^ fluorimeter quartz cuvette, was excited either at 420 nm by the built-in second harmonic output of a mode locked Ti:sapphire laser (Tiger-ps SHG, Time Bandwidth Products, Zurich, Switzerland) or at 355 nm, by the third harmonic of a mode-locked Nd:VAN laser (GE-100, Time Bandwidth Products). The TH was obtained out of cavity as described in [53]. The fluorescence at λ > 450 nm (λ > 400 nm for the measurements with excitation at 355 nm) was collected at 90° to the excitation beam through cut-off filters (LL-450 or LL-400, respectively, Corion, Holliston, MA, USA) by a 20× microscope objective and focused onto the sensitive area of a PDM50 single-photon avalanche diode (Micro-photon-devices, Bolzano, Italy). All fluorescence decays were collected up to >10,000 peak counts in strict single photon regime by suitably attenuating the excitation beam with neutral-density filters. The maximum absorbance of the solutions at the excitation wavelength was 0.1.

The fluorescence decay data were fitted, without performing deconvolution of the system pulse response, to either double, triple or four-exponentials above a constant background, by minimizing the chi-square value through a Levenberg-Marquardt algorithm. The number of exponential components was established by adding, one by one, exponential components to the fitting function until the fitting routine converged to yield two components of equal time constant. The means of the values obtained from the fits of experiments performed on three parallels, with errors given by the standard deviations, were assumed as the time constant, τ*_i_*, and initial amplitude, A*_i_*, of the *i*-th decay component, being the A*_i_* values calculated at the peak channel of the experimental data.

### 3.5. Photodegradation Quantum Yields and Half-Lives

The photodegradation quantum yield of CYV in selected solvents was measured using the potassium ferrioxalate chemical actinometer [57]. The samples were irradiated at 387 ± 10 nm by using a monochromator (Applied Photophysics Ltd., Leatherhead, UK, f 3.4, 900 W xenon arc lamp). The number of sample molecules reacted per unit time and per unit volume as a function of exposure time was quantified by means of reversed phase HPLC. The separation was performed on a 150 × 3.9 mm Nova Pak^®^ C_18_ column (Waters, Milford, MA, USA). The mobile phase was a mixture of ACN and 0.5% citric acid buffer, adjusted to pH 3 with KOH (ACN/buffer 35:65). The samples were detected at 350 nm. The chromatic system consisted of a LC-9A pump, a SP D-10A UV-VIS detector, a SIL-10 DV auto sampler and a C-R3A integrator (Shimadzu, Kyoto, Japan).

The photodegradation half-lives were measured in the Suntest CPS (Heraeus, GmbH, Hanau, Germany). The light source was a xenon lamp (1.8 kW) equipped with a glass filter (with cut-off at approximately 310 nm). The irradiation was measured to be 1.4 × 10^5^ lux and 14.8 W/m^2^ in the visible and UV range, respectively, by use of a lux meter in combination with a filter radiometer (Hagner ECI Digital luxmeter, Hagner ECI UV-A, Hagner, Solna, Sweden). The samples were irradiated in quartz cuvettes under continuous stirring. The changes in CYV concentration with exposure time was monitored by HPLC as described above. The observed first-order rate constants for the degradation were obtained from linear regression analysis of the logarithm of the CYV concentration plotted against time. The calculated rate constants were corrected for the difference in absorptivity (*i.e.*, area under the absorption curve) between the various samples. The photodecomposition half-life was calculated from the rate constant by use of the first-order kinetic model. Both the quantum yield and half-life measurements were carried out in triplicate.

### 3.6. Isolation of Photodecomposition Products

A sample of CYV in ethanol (1 mg/mL) was exposed to radiation for 15 min in a 500 mL photoreactor equipped with a lamp TQ 150 (240–600 nm). A glass filter was inserted to avoid radiation below 310 nm. The samples were evaporated to dryness under vacuum after exposure. The dried samples were dissolved in 1 mL ethanol and separated by TLC (stationary phase: silica with fluorescence indicator; mobile phase: chloroform/ethanol 25:1). After separation the individual degradation products were eluted from the silica by dilution in ethanol.

### 3.7. Spectrofluorimetric Detection of Photosensitized Reactive Oxygen Species Generation

The production of ROS by CYV was compared to that of CURC in methanol by using the fluorescent indicator diphenylisobenzofuran. Solutions at 5 μM photosensitizer (*i.e.*, CURC or CYV) concentration and 50 μM concentration of diphenylisobenzofuran were prepared and divided in aliquots which were stored in the dark during the experiment. Blank solutions without diphenylisobenzofuran were also prepared. Each aliquot was irradiated for a predetermined time, spanning from s to 10 min, by the PTI spectrofluorimeter lamp set at 85 W power, in a 20 nm band around the photosensitizer’s absorption peak (*i.e.*, 423 nm for CURC and 385 nm for CYV). Then, the excitation lamp shutter was closed to block photosensitization, the excitation bandwidth was changed to 1 nm, and the fluorescence of diphenylisobenzofuran was measured in the band 430–600 nm upon excitation at 415 nm. Similar procedures were applied to the blanks, and the fluorescence of CURC and CYV (and its degradation products) was subtracted. The fluorescence intensity at the 475 nm emission peak of diphenylisobenzofuran was plotted *versus* light-exposure time in order to assess the amount of generated *o*-dibenzoylbenzene. A fresh aliquot of both sample and blank was used to measure the ROS production after each specific irradiation time.

## 4. Conclusions

The curcumin derivative cyclovalone promises superior excited-state stability and preserved biochemical reactivity with respect to the parent compound by virtue of its molecular structure in which the β-diketo core is substituted by a saturated cyclohexanone ring unable to undergo highly destabilizing excited-state proton transfer reactions, while the phenyl groups are conserved. The compound was evaluated as to its H-bonding properties, excited state dynamics, photostability and photosensitized reactive oxygen species production efficiency. As expected the average decay times measured for cyclovalone in all the considered solvents were much longer than the corresponding values measured for curcumin. Longer lived excited states might lead to an increased phototoxic potential of optimized pharmaceutical formulations based on this active principle. However, the compound evidenced a severe tendency to photodegradation in most of the tested environments.

## Supplementary Materials

Supplementary materials can be accessed at: http://www.mdpi.com/1420-3049/19/9/13282/s1.

## Supplementary Files

Supplementary File 1
