# Supplementary material for: Elucidation of the Relationships between H-Bonding Patterns and Excited State Dynamics in Cyclovalone
Source: Molecules. 2014 Aug 28;19(9):13282–304. doi: 10.3390/molecules190913282 (PMC6270912; doi:10.3390/molecules190913282)

Supplementary Materials

Figure S1. Absorption spectra of CYV solutions in ethanol straight after preparation (see "Fresh sample" spectrum) and after illumination in the Suntest CPS, under the experimental conditions fully described in Materials and Methods, for up to 21 min, in intervals of 3 min.

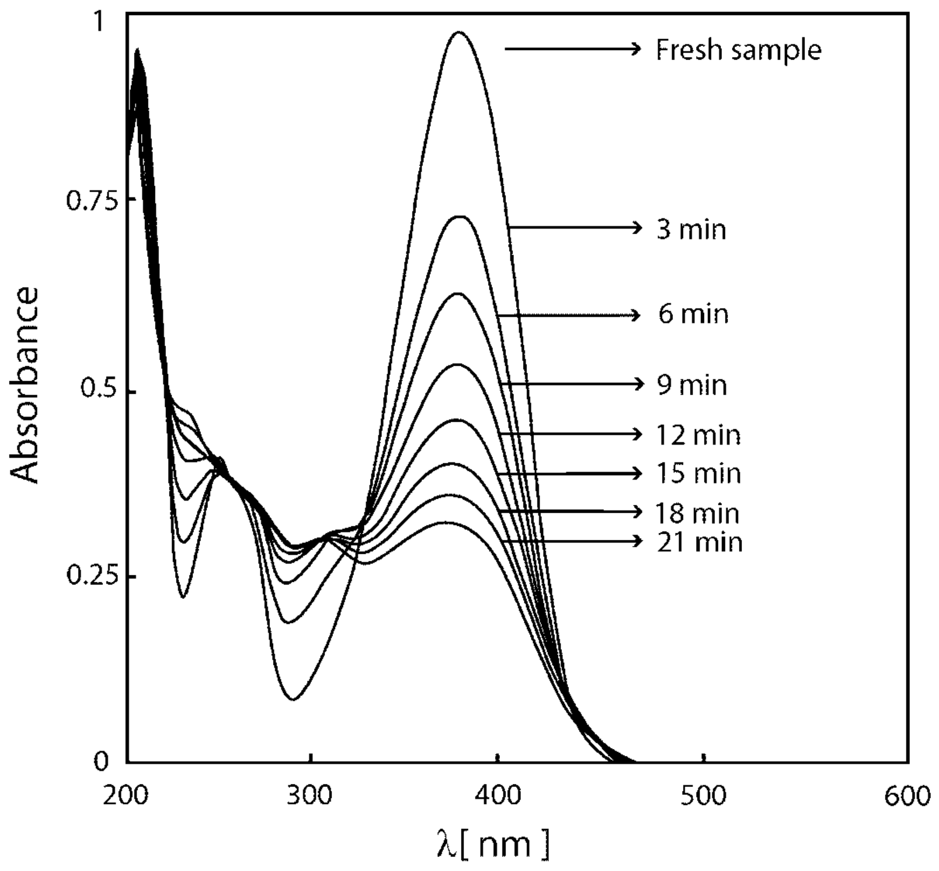

Figure S2. Absorption spectra of CYV and the five degradation products isolated by TLC in an ethanol solution previously submitted to illumination in the Suntest CPS, under the experimental conditions fully described in Materials and Methods, for 15 min. In the figure inset sketch of the relative position of the compounds line on the TLC plate.

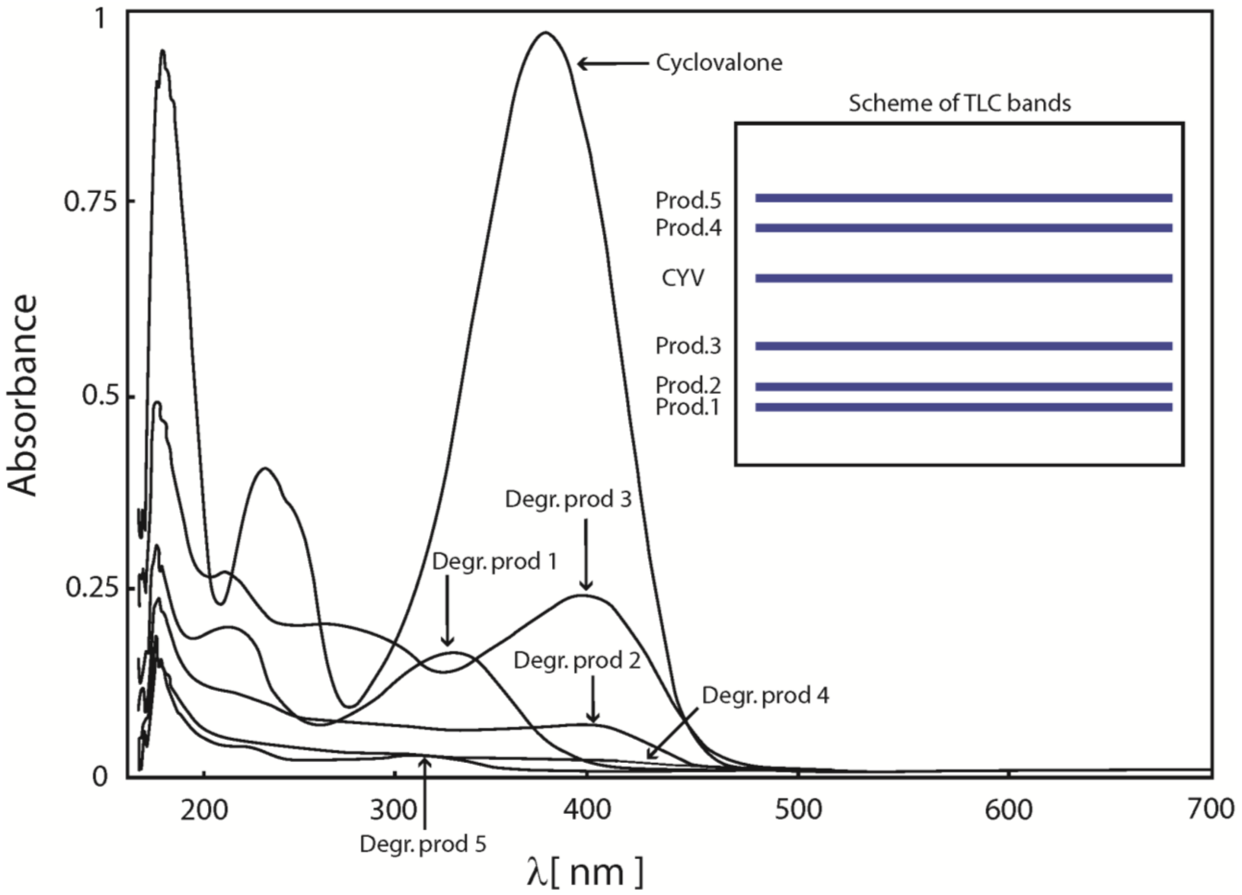

Supplement: Supplementary File 1 [file molecules-19-13282-s001.pdf]
